# Low-Field NMR Relaxometry for Intraoperative Tumour Margin Assessment in Breast-Conserving Surgery

**DOI:** 10.3390/cancers13164141

**Published:** 2021-08-17

**Authors:** Valeria Bitonto, Maria Rosaria Ruggiero, Alessandra Pittaro, Isabella Castellano, Riccardo Bussone, Lionel M. Broche, David J. Lurie, Silvio Aime, Simona Baroni, Simonetta Geninatti Crich

**Affiliations:** 1 Department of Molecular Biotechnology and Health Sciences, University of Torino, 10126 Torino, Italy; valeria.bitonto@unito.it (V.B.); mariarosaria.ruggiero@unito.it (M.R.R.); silvio.aime@unito.it (S.A.); simonetta.geninatti@unito.it (S.G.C.); 2 Pathology Unit, Department of Medical Sciences, University of Turin, 10126 Torino, Italy; apittaro@cittadellasalute.to.it (A.P.); isabella.castellano@unito.it (I.C.); 3 Breast Unit, Ospedale Cottolengo, 10152 Torino, Italy; riccardo.bussone@ospedalecottolengo.it; 4 Aberdeen Biomedical Imaging Centre, University of Aberdeen, Foresterhill, Aberdeen AB25 2ZD, UK; l.broche@abdn.ac.uk (L.M.B.); d.lurie@abdn.ac.uk (D.J.L.); 5 IRCCS SDN, Via E. Gianturco 113, 80143 Napoli, Italy

**Keywords:** low-field NMR relaxometry, breast cancer, margin assessment, breast-conserving surgery, cross-membrane water exchange

## Abstract

**Simple Summary:**

Breast cancer is the most diagnosed cancer for women, and clear surgical margins in breast-conserving surgery (BCS) are essential for preventing recurrence. In this study, the potential of fast field-cycling ^1^H-NMR relaxometry as a new tool for intraoperative margin assessment was evaluated. The technique allows the determination of the tissue proton relaxation rates as a function of the applied magnetic field on small tissue samples excised from surgical specimens, at the margins of tumour resection, prior to histopathological analysis. It was found that a good accuracy in margin assessment, i.e., a sensitivity of 92% and a specificity of 85%, can be achieved. The discriminating ability shown by the relaxometric assay relies mainly on the difference of fat/water content between healthy and tumour cells. The information obtained has the potential to support the surgeon in real-time margin assessment during BCS.

**Abstract:**

As conserving surgery is routinely applied for the treatment of early-stage breast cancer, the need for new technology to improve intraoperative margin assessment has become increasingly important. In this study, the potential of fast field-cycling ^1^H-NMR relaxometry as a new diagnostic tool was evaluated. The technique allows the determination of the tissue proton relaxation rates (*R*_1_), as a function of the applied magnetic field, which are affected by the changes in the composition of the mammary gland tissue occurring during the development of neoplasia. The study involved 104 small tissue samples obtained from surgical specimens destined for histopathology. It was found that a good accuracy in margin assessment, i.e., a sensitivity of 92% and a specificity of 85%, can be achieved by using two quantifiers, namely (i) the slope of the line joining the *R*_1_ values measured at 0.02 and 1 MHz and (ii) the sum of the *R*_1_ values measured at 0.39 and 1 MHz. The method is fast, and it does not rely on the expertise of a pathologist or cytologist. The obtained results suggest that a simplified, low-cost, automated instrument might compete well with the currently available tools in margin assessment.

## 1. Introduction

Breast cancer is the most commonly diagnosed cancer and the leading cause of cancer death for women worldwide [1]. The recommended treatment for early-stage disease is breast-conserving surgery (BCS) [2]. This procedure seeks to remove cancer, leaving a margin of healthy tissue surrounding the excised specimen while providing a satisfactory cosmetic outcome. For many years, numerous trials have demonstrated equivalent survival outcomes for mastectomy and for breast-conserving therapy if clear margins are maintained during the surgery [3]. A surgical margin is defined as negative if no malignant cells are observed at the outer layer of the resected specimen for invasive and in situ cancers [4]. The presence of tumour cells at the surgical margin is the strongest predictor of locoregional recurrence, resulting in a two-fold increased risk of recurrence.

The current gold standard for margin classification is provided by the analysis conducted by the surgical pathologist, carried out on the specimens after surgery. The tissue, removed by the surgeon, is fixed, processed, sectioned, stained with hematoxylin and eosin (H&E), and analysed by optical microscopy. The method is robust and accurate, but it is time consuming, as it usually takes several days, depending on the specimen size and diagnostic complexity. During the macroscopic evaluation of the specimen, the pathologist applies coloured inks on the tissue surgical margins in order to recognise the true margin [4].

If positive or closely contiguous margins are found, then a second surgical procedure may be needed. About 20–40% of BCS procedures result in margins, which are either positive or suspected of having malignant cells at the margins of the resection region [5]. A return to the surgery room for margin re-excision results in additional anxiety for patients; delays in the onset of adjuvant therapy; additional exposure to the risks of anaesthesia; increased surgical complications, including increased surgical site infections; lower patient satisfaction; lower rates of cosmetic acceptability; increased health care costs; and even increased probability to go for bilateral mastectomies [6,7,8]. On this basis, it is evident that a clinical need exists calling for methods able to support the surgeon’s decisions on the identification of tumour margins during BCS.

Several intraoperative margin assessment strategies have been proposed to reduce the need for re-excision, but they all have significant clinical and technical limitations that have hampered widespread adoption [9,10]. As the need for fast and accurate methods for the analysis of surgical margins is well recognised as a critical factor for successful BCS, an active search for the identification of suitable techniques is still ongoing. The target would be the identification of a rapid, low-cost, easy-to-use method that is able to detect malignancy at the surface of the excised tissue while keeping false positive margins low to avoid removing excess tissue (i.e., a method with a sensitivity above 90% and a specificity near 85%) [9]. Among the methods recently proposed, intraoperative flow cytometry has emerged as a promising technique to be used not only in BCS but also for intracranial tumour surgery [11,12]. The main disadvantage of the technique is due to the preliminary sample processing that may not be fully reproducible. Many methods using fluorescent dyes injected into the patient or incubated with the tissue specimen are under development [13,14,15,16,17,18], showing high sensitivity and specificity but restricted to surface imaging with low tissue penetration and requiring a long process for the identification and authorisation of the proper candidate for a given tumour. Quantitative micro-elastography (QME) is an emerging technique that produces images of tissue microscale elasticity and has also been recently proposed for margin assessment in cancers that are known to exhibit altered mechanical properties [19], but the difficult interpretation of the images obtained remains the main drawback for this technology. Intraoperative ultrasound guidance of excision [20] has also received a lot of attention over the years but is strongly limited by operator-dependent outcomes. Other approaches use the radiofrequency electric properties of tissues [21], with a commercial system currently available, but show poor results when dealing with multiple tissue types within a region. Mass spectrometry [22] has also shown interesting capabilities to map changes in concentration of substrates associated with the tumour metabolism; however, it is a destructive method.

Magnetic resonance imaging (MRI) at high magnetic fields, which noninvasively provides detailed images of deep tissues with a very high spatio-temporal resolution, is widely used for cancer detection in diagnostic departments. Contrast-enhanced MR images acquired preoperatively are used for surgical planning. The diagnostic information is excellent, and they often allow the detection of small satellite lesions [23,24,25]. Unfortunately, the supportive capabilities of MRI cannot be translated to guide the tumour resection directly in the operating theatre for real-time margin assessment because intraoperative MRI is a highly technologically demanding technique that is rarely employed. Recently, a mobile MRI scanner (ClearSight^TM^ system) has been proposed for tumour margin detection that can be used in the surgery room or nearby for tissue specimen analysis [26]. The diagnostic response obtained on the resected tissue is based on diffusion-weighted (DWI)-MRI, a technique widely used in the diagnosis of most solid cancers. DWI-MRI is sensitive to tissue cellularity and malignancy, specifically for solid tumours, such as breast cancer [27,28], and was reported to show high sensitivity, specificity, and accuracy (91%, 93%, and 92%, respectively). However, being based on MRI scanners, the method requires a complex and expensive technology with the use of a sophisticated algorithm for data processing as well as skilled operators for the acquisition and interpretation of the results.

The herein reported results show that an approach based on nuclear magnetic resonance (NMR) relaxometry may allow tumour margin assessment on excised tissue specimens without the need for spatial discrimination (i.e., without imaging). It is well known that proton *T*_1_ values of a given tissue decrease as a function of the applied magnetic field strength, thus providing larger differences among the tissues the lower the applied magnetic field strength is [29,30,31]. This behaviour, known as *T*_1_-dispersion, is closely linked to the mechanisms that drive molecular dynamics within the sample and can therefore be exploited in the search for novel diagnostic markers. Indeed, fast field-cycling (FFC)-NMR instruments are readily available and can measure *T*_1_ dispersion by switching (cycling) the magnetic field between different field strengths during the measurement procedure [32], overcoming the problem of low sensitivity associated with low fields while allowing a relatively rapid acquisition of *T*_1_ at various magnetic fields. The curve produced is called a *T*_1_ nuclear magnetic relaxation dispersion (NMRD) profile or, equivalently, an *R*_1_ NMRD profile where *R*_1_ = 1/*T*_1_. FFC-NMR relaxometers are extensively used in research laboratories for the characterisation of materials and experimentation and usually exploit signals provided by the most abundant proton-containing components in the system under investigation, often represented by water and fat in biological specimens. FFC-NMR relaxometers rely on the same principles as NMR spectrometers and MRI scanners, but the technical solutions involved are relatively simpler and significatively less expensive, offering the potential for more widespread take-up of the technology in the intended application.

An important advantage of this approach is that it does not need exogenous contrast agents to obtain functional information. It has been shown by our group that NMRD profiles can act as a high-sensitivity tool for cancer detection and staging in ex vivo murine breast tissues collected from Balb/NeuT mice [33] or in breast cancer cell lines [34] and in vivo with mice transplanted with murine mammary cancer cells (4T1, TS/A, 168Farn) [35].

In the work described here, this methodology is employed to assess the presence of tumour cells in small tissue samples excised from surgical specimens, at the margins of tumour resection, prior to histopathological analysis. Relaxation at low magnetic field strengths is affected by the changes in the composition of the mammary gland tissue (lipids/proteins/water) occurring during the development of neoplasia. The information obtained has the potential to support the surgeon in real-time margin assessment during breast-conserving surgery.

## 2. Materials and Methods

### 2.1. Collection of Human Breast Cancer Samples

The study was approved by the Research Ethics Committee for Human Biospecimen Utilization (Department of Medical Sciences—ChBU) of the University of Turin. Written consent was not required considering the retrospective nature of the study. All cases were de-identified, and all clinical-pathological data were accessed anonymously. The study was conducted in accordance with The Code of Ethics of the World Medical Association (Declaration of Helsinki).

Forty-one patients (40 females and 1 male aged between 40 and 90 years, mean age 65 ± 15 years, median 63 years) undergoing lumpectomy/mastectomy for breast cancer at Cottolengo Hospital in Turin, Italy, were enrolled in the study. Lumpectomy/mastectomy specimens were placed under vacuum immediately after excision in the surgery room, stored at 4 °C, and then transferred on ice to the histopathology laboratory within 24 h. Before fixing tissues for routine histopathological analysis, from each of the lumpectomy/mastectomy specimens, two or three samples (weight between 16 and 114 mg (2.5–8.9 mm Ø)) were resected, leading to a total of 104 freshly excised breast tissue samples. The samples were resected from different areas of the surgical specimen by the pathologist, following an on-site, real-time, macropathology gross examination to provide a variety of tissue types. *R*_1_ measurements were acquired within 1 h from the sampling. During transfer and waiting time, samples were stored on ice, and *R*_1_ measurements were carried out at 10 °C to preserve tissue integrity for further histopathological analysis as described below.

### 2.2. Relaxometric Characterisation

Tissue samples were weighted and placed into a 5 mm Ø capped glass tube, 9 mm long. The acquisition of proton longitudinal relaxation time (*T*_1_) as a function of the magnetic field strength (the NMRD, profile) was performed on a Stelar SpinMaster FFC-NMR relaxometer (Stelar S.n.c., Mede, Italy), equipped with a signal-detection microcoil of 10 mm diameter, at 10 °C in the 0.02–1 MHz proton Larmor frequency (PLF) range (corresponding to applied magnetic field *B*_0_ = 0.48 mT–24 mT). The overall acquisition time of the NMRD profile (6 magnetic field points) was 17′48″. A pre-polarised sequence was used [36] with pre-polarisation at 25 MHz and detection at 14.5 MHz, a field switching time of 4 ms, a 90° pulse length of 5.5 μs, and 32 incremented relaxation delays (see details in Table 1 below). The relaxometer operated under complete computer control with a relative uncertainty in the 1/*T*_1_ value of ± 2%, calculated considering the pooled standard deviation (*S*_p_). The analysis of the magnetisation decay curves (*M*_z_) for the determination of *T*_1_ was carried out using the commonly adopted mono-exponential fitting procedure of the experimental data to the curve, calculated on the basis of the Bloch equations.

The reference NMRD profiles of adipose and tumour tissue were obtained by averaging the profiles of healthy (H) samples containing at least 90% adipose tissue (*n* = 17) and tumour (T) samples containing at least 70% tumour tissue (*n* = 7), respectively. Among them, the outliers (4 H and 1 T, associated with mucinous-type tumour) were excluded. The outliers were samples displaying *R*_1_ values significantly far from the average, i.e., above or below the fences (defined as the 75th Percentile +/− (1.5 × Interquartile Range)).

### 2.3. Reproducibility of T*_1_* Measurements

FFC NMR is a relatively time-consuming technique, as it is not possible to repeat a statistically high number of observations with the same fresh tissue sample. In order to assess the repeatability of the protocol, the NMRD profiles of 9 tissue samples (6 samples in the first session and 3 samples in the second one, respectively) of different weight (from 28 to 126 mg) were measured at 1 MHz and 0.02 MHz in triplicate, under identical conditions. For each triple of measurements at the same magnetic field strength, the pooled standard deviation *S*_p_ was calculated:

(1) $$S_{p}=\;\sqrt{\frac{S_{1}^{2}+\;S_{2}^{2}+\ldots +S_{k}^{2}\;}{k}}$$

where *S_k_* is the within-sample standard deviations and *k* is the number of samples (*k* = 9) [37].

### 2.4. Histology Characterisation

After the acquisition of the NMRD profiles, tissue samples were fixed in formalin 10% and embedded in paraffin. All the specimens were serially sectioned at 5 μm thickness to analyse the tissue sample entirely. H&E tissue slides were scanned using the Pannoramic DESK II DW (3DHistech), then the different tissue components present in the sample (i.e., fat, stroma, and tumour) were quantified. ROIs containing normal breast tissue (fat, glandular, and fibrous tissue) and tumour were manually drawn and were used for quantification of the different tissue components presents in the sample. Image analysis was performed using ImageJ 1.53c software [38].

## 3. Results

**Figure 1:**
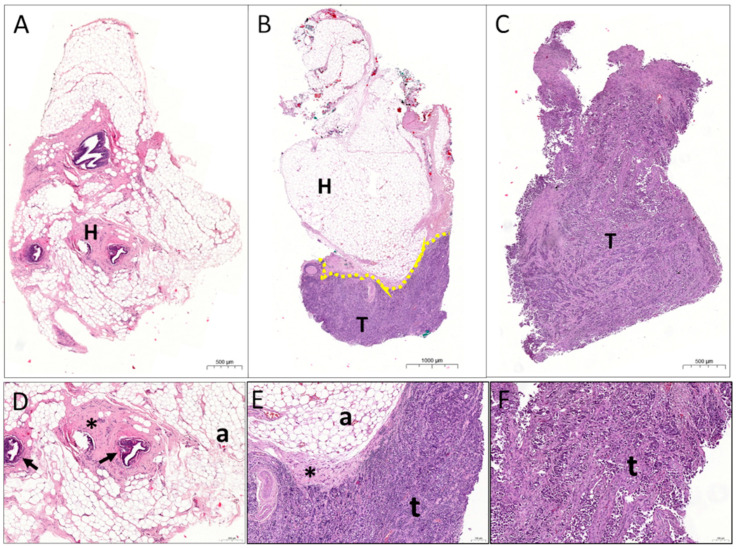
Representative images of healthy (**A**), mix (**B**), and tumour (**C**) breast tissue samples stained by H&E. (**D**–**F**) magnification of (**A**–**C**), respectively. Arrows indicate normal mammary ducts, * indicate fibrous tissue, “a” indicate adipocytes, and “t” tumour cells.

This study involved the analysis of 104 small-sized breast tissue samples (with a range of 2.5–8.9 mm Ø) cut from quadrantectomy or mastectomy surgical specimens. The histological analysis performed by E&H staining (Figure 1) allowed classification of the specimens investigated as follows: 40 healthy (H), 21 tumours (T), and 43 containing a mixture of both (M). The histopathological analysis of tissue samples containing tumour revealed the presence of invasive and in situ carcinoma with varying abundance.

**Figure 2:**
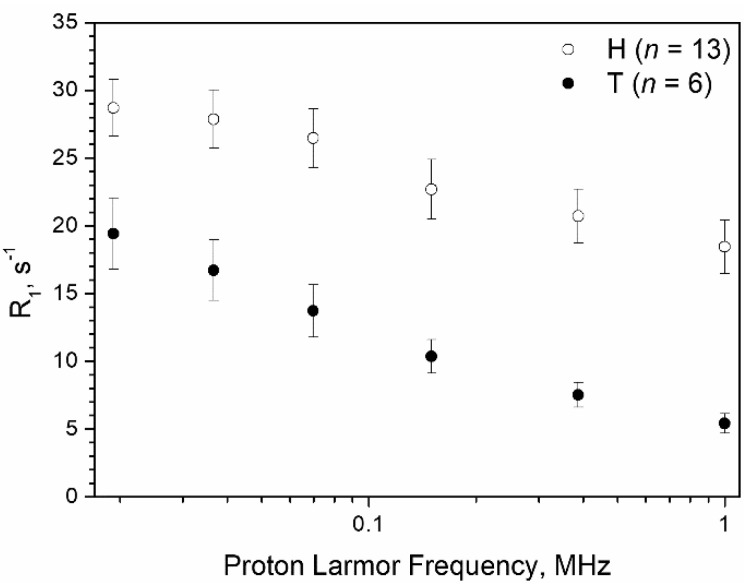
Comparison between typical *R*_1_ NMRD profiles of healthy specimens (H, *n* = 13) and tumour samples (T, *n* = 6). Error bars represent the SD.

Figure 2 reports typical NMRD profiles of H samples (*n* = 13) and T samples (*n* = 6), excluding outliers (see above). In both H and T samples, the remaining tissue corresponded to stroma and/or stroma + adipose tissue.

As shown in Figure 2, both H and T tissue relaxation rates increase when the magnetic field strength decreases, but the relative values and slopes of the two curves are significantly different. This finding appears to be associated, first of all, with the different water content and water mobility characteristics of the tissues: tumour tissue has a protein/fat/water content that is highly altered with respect to healthy breast tissue, in which adipocytes are dominant and lipids account for up to 70–80% of tissue content. As previously reported [33,39], lipid proton relaxation rates show significatively less dispersion with the magnetic field strength in the range 0.02–10 MHz than water/protein protons. Therefore, relaxation rates of H tissues show higher values and a less pronounced dispersion with the magnetic field with respect to T tissues (Figure 2). The *T*_1_ measurements showed a good reproducibility (±2%), expressed as the pooled standard deviation (*S*_p_) calculated as described in Section 2.3. Accordingly, we defined two relaxometric quantifiers that captured this particular behaviour and allowed the assessment of the presence of tumour cells in a breast tissue specimen. The first one was the ratio between the *R*_1_ value measured at the lowest (0.02 MHz) and highest (1 MHz) magnetic fields, i.e., *R*_1_^0.02MHz^/*R*_1_^1MHz^, later referred to as the Ratio. The second one was the sum of the *R*_1_ values measured at 0.39 MHz and 1 MHz, later referred to as the 2R_1_ value. The receiver operating characteristic (ROC) curve analysis was then used to assess the performance of each of the two quantifiers in the discrimination among negative (H) and positive (M + T) specimens. The ROC is commonly employed to assess the cost/benefit analysis of diagnostic decision making.

**Figure 3:**
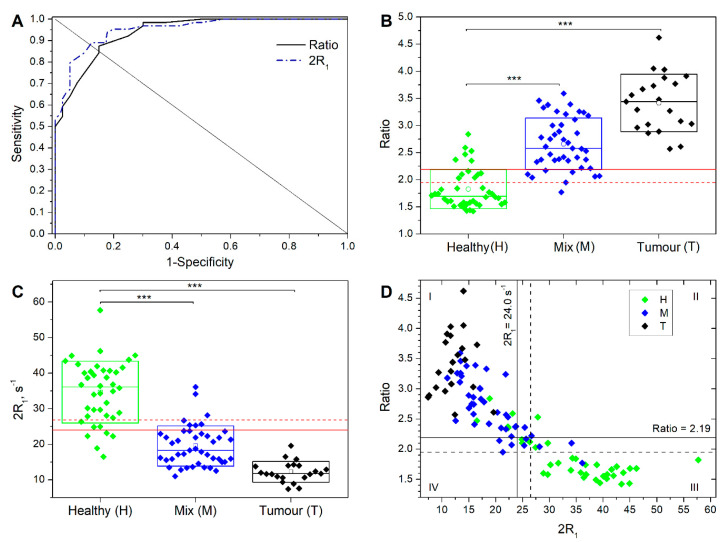
(**A**) ROC curve for the dataset describing the sensitivity vs. 1 minus specificity for different Ratio and 2R_1_ cut-off values plotted against the equal error rate line where there is an equal probability of miss-classifying a positive or negative sample. (**B,C**) Box chart of the Ratio and 2R_1_ values calculated for the 104 investigated tissue samples. The box is determined by the mean ± SD. The line inside each box represents the 50th percentile (median). The thick line represents the cut-off value (see text). Statistical significance was determined by Student’s *t*-test (*** *p* < 0.01). (**D**) The Ratio value as a function of the 2R_1_ value for all the 104 samples, labelled by colour as H, M, and T.

Figure 3A shows the ROC curve analysis describing the sensitivity versus 1 minus specificity for different Ratio and 2R_1_ cut-off values. Using the entire range of the calculated Ratio (1.42–4.62) and 2R_1_ (7.42–57.7), the best cut-off values were found to be 2.19 and 24.0 s^−1^, respectively. The calculated area under curve (AUC) is an index of accuracy and was 0.95 for both criteria.

In particular, the Ratio cut-off value of 2.19 provided a sensitivity, specificity, and accuracy of 88%, 85%, and 85%, respectively; 8 M and 6 H specimens were misassigned giving rise to eight false negative (FN) and six false positive (FP) samples, respectively (Figure 3B). The mean (±SD) Ratio calculated for each group of specimens was 1.83 ± 0.36, 2.66 ± 0.48, and 3.42 ± 0.53 for H, M, and T, respectively. The averaged Ratio found for H specimens was significantly different from that observed for both T and M tissues (*p* = 3.63 × 10^−20^ and 1.18 × 10^−13^, respectively).

Similarly, the 2R_1_ cut-off value of 24.0 s^−1^ provided a sensitivity, specificity, and accuracy of 89%, 88%, and 88%, respectively; 7 M and 5 H specimens were misassigned, giving rise to seven false negative (FN) and five false positive (FP) samples, respectively (Figure 3C). The mean (±SD) 2R_1_ calculated for each group of specimens was 34.7 ± 8.7, 19.5 ± 5.7, and 12.2 ± 2.9 for H, M, and T, respectively. The averaged 2R_1_ found for H specimens was significantly different from that observed for both T and M tissues (*p* = 1.40 × 10^−16^ and 9.68 × 10^−15^, respectively).

Figure 3D reports the Ratio value as a function of the 2R_1_ value for all the 104 samples, labelled as H, M, and T. The two identified cut-off values correspond to the horizontal and vertical thick lines, giving rise to four different quadrants. It is possible to notice that most of the T and M specimens (84%) fell into quadrant I (above the Ratio and below the 2R_1_ values, respectively) and most of the H specimens (85%) in the quadrant with the opposite characteristics (quadrant III: below the Ratio and above the 2R_1_ values, respectively). The few samples (8.4% compared to the total of samples) that respect only one of the inequalities with respect to the cut-offs fell in the remaining quadrants (II: above Ratio and above 2R_1_ values, respectively; IV: below Ratio and below 2R_1_ values, respectively).

Due to the high similarity found in the performance of the Ratio and 2R_1_ criteria and with the aim of developing a method that is at the same time rapid and accurate for the intraoperative margin assessment, we considered a two criteria protocol in which the verification with the second criterion (and therefore the execution of an additional measure) is necessary only for a small number of borderline samples.

**Figure 4:**
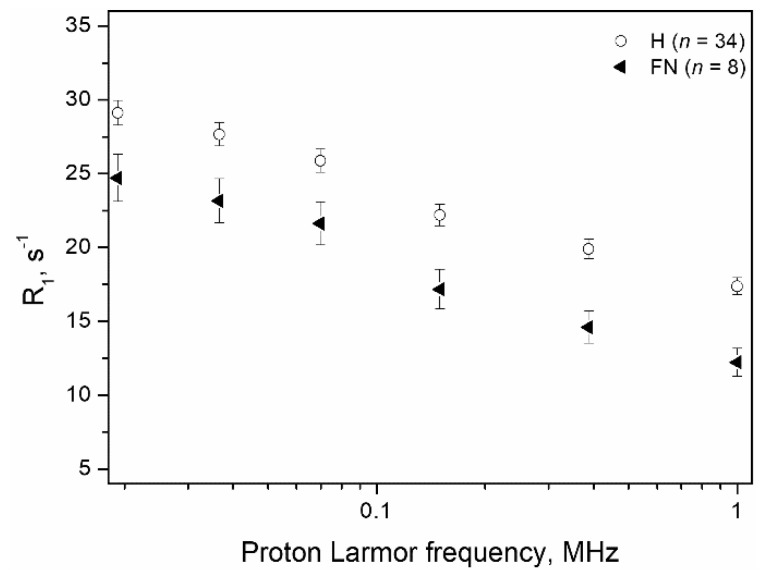
Comparison between the NMRD profile of the H samples with the averaged NMRD profiles of the 8 specimens that, on the basis of the applied Ratio criterion, resulted to be FN samples (n = 8). Error bars represent the standard errors.

By applying this procedure, the Ratio was selected as the first criterion in sample discrimination. Then, only the samples with a Ratio between 1.95 ≤ Ratio ≤ 2.19 (6 H and 7 M) were considered for the second criterion (2R_1_). The rationale relies on the observation that in this area fell misassigned M specimens containing a significant amount of adipose tissue (58.4% ± 8.6% (SE)), and this type of composition affects the Ratio more than the 2R_1_ parameter (Figure 4). Figure 4 shows that the averaged NMRD profile of the FN samples diverged more from the trend of the correctly assigned H tissues (*n* = 34) at high magnetic field strengths, showing lower *R*_1_ values (the calculated p values were 0.02513 at 0.02 MHz and 0.00029 at 1 MHz, respectively). Moreover, the two-criteria protocols improve sensitivity, favouring it over specificity, respecting the prevailing diagnostic importance of avoiding false negative (FN) margins. In this way, the H sample assignment was confirmed, and three of the FN samples (the samples in quadrant IV) could now be correctly assigned. Accordingly, a sensitivity, specificity, and accuracy of 92%, 85%, and 89%, respectively, were achieved.

Repeating the analysis using 2R_1_ as the first selection criterion, the samples with a 2R_1_ value between 24.0 s^−1^ ≤ 2R_1_ ≤ 26.5 s^−1^ (3 H and 3 M) were subjected to the Ratio analysis criterion. The correct assignment of the H samples was confirmed, and one out of the two M samples in quadrant II was then correctly assigned (−1 FN). A sensitivity, specificity, and accuracy of 91%, 88%, and 89%, respectively, were finally achieved.

**Figure 5:**
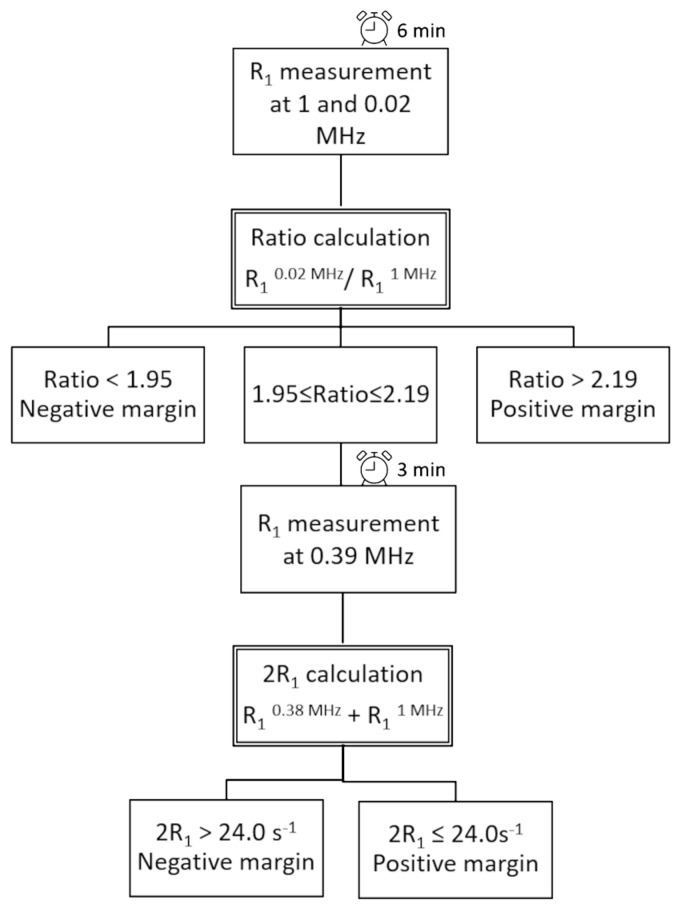
Schematic representation of the experimental procedure to evaluate margins from excised specimens.

Figure 5 summarises the protocol developed here, which consists of the following steps:

1. Measurement of *R*_1_ at 0.02 MHz and 1 MHz (total time needed: 6 min);
2. Calculation of the Ratio;
3. Assigning samples with Ratio > 2.19 as positive and Ratio < 1.95 as negative;
4. For samples showing a Ratio between 1.95 and 2.19, a further *R*_1_ acquisition at 0.39 MHz is performed (total time needed: 3 min);
5. Calculation of 2R_1_;
6. Assigning samples showing a 2R_1_ > 24.0 s^−1^ as negative and 2R_1_ ≤ 24.0 s^−1^ as positive.

The total time needed to perform the complete specimen assessment protocol is about 9 min. Processing time is negligible, and improvements in the FFC-NMR acquisition protocol may significantly reduce the duration of the overall analysis.

## 4. Discussion

In this work, we focused on the application of low-field relaxometry as a technique to tackle the important clinical need for the assessment of tumour margins during surgery. The proposed method relies on the determination of two quantifiers, namely (i) the slope of the line joining the *R*_1_ values measured at 0.02 and 1 MHz (Ratio) and (ii) the sum of the *R*_1_ values measured at 0.39 and 1 MHz. The latter value integrates the main discriminating parameter associated with the slope measured over largely different magnetic fields because it was noticed that the *R*_1_ values of H tissue were similarly high, whereas the corresponding values for T specimens were significantly different. When their relative contribution is brought to the M specimens, they become the determinants for the definitive assignment. We developed a method that privileged higher sensitivity over specificity by applying two criteria in a specific order (Ratio first, 2R_1_ second). The sensitivity (92%) and specificity (85%) attained appear competitive with other methods and well comparable with the best procedures currently proposed for intraoperative tumour margin assessment [9,10].

FFC relaxometry appears to be a suitable technique to assess the presence of tumour cells in excised tissue specimens. In the case of the breast tissues considered in this work, the approach proposed relied on the large differences in the relaxometric response shown by healthy and tumour tissues, as the former is rich in lipid-containing adipocytes. It is also likely that the amount of water and, even more importantly, the exchange rate of water molecules across the cellular membranes both have a role in the differentiation of T from H tissues [35]. Cross-membrane water exchanges are directly related to cellular metabolism and may have a dominant role in other types of tumours [40]. In any case, the increased exchange rate of water molecules across the cellular membrane should have a similar effect on the NMRD profiles than the decrease in lipid content and could be useful to gain more insight into tumour aggressiveness since the inter-compartment water dynamics is accelerated by enhanced cellular metabolism.

An important aspect of the relaxometric method is that it is carried out without requiring any additional treatment of the excised specimen and only needs a reasonable time to complete (currently 6–9 min), which is well compatible within the timeframe of BCS. This method can be applied by introducing a minor modification to the procedure currently applied during the transfer to the pathology laboratory. The present study was carried out using a commercial FFC relaxometer that is not mobile and has a relatively small sample holder, but one may anticipate that specialised instrumentation may be developed for this particular application, including automatised procedures. While a small-sample approach is also used in other approaches for tumour margin assessment [41], large-area scanning capabilities may also be explored using surface coils to acquire the proton NMR signal down to 1–2 mm from the surface.

## 5. Conclusions

We believe that the results reported here clearly indicate that tissue relaxometric data obtained at different magnetic field strengths may provide a useful tool to assess the presence of tumour cells in an excised tissue specimen by a simple quantitative analysis that does not need highly specialised operators for the interpretation of the data obtained.

## Figures and Tables

**Table 1:** Parameters of NMRD profile acquisition.

Evolution Field (MHz PLF)	Range of Evolution Time (s)	Time per Field	Distribution
0.02, 0.037, 0.07	0.01 to 2.8	2′46″	Log
0.15, 0.39, 1	0.01 to 4	3′10″	Log

## Data Availability

The main research data supporting the results of this study are included in Figure 1, Figure 2, Figure 3 and Figure 4. Other data can be made available upon reasonable request from the corresponding author.
